# Detection of dairy fouling by cyclic voltammetry and square wave voltammetry

**DOI:** 10.1002/fsn3.1463

**Published:** 2020-03-16

**Authors:** Olga Fysun, Sara Khorshid, Johannes Rauschnabel, Horst‐Christian Langowski

**Affiliations:** ^1^ TUM School of Life Sciences Weihenstephan Technical University of Munich Freising Germany; ^2^ Robert Bosch Packaging Technology GmbH Waiblingen Germany; ^3^ Department of Mechanical and Process Engineering University of Kaiserslautern Kaiserslautern Germany; ^4^ Fraunhofer Institute for Process Engineering and Packaging IVV Freising Germany; ^5^ Present address: Robert Bosch GmbH Reutlingen Germany; ^6^ Present address: Sanofi‐Aventis Deutschland GmbH Frankfurt Germany

**Keywords:** cyclic voltammetry, dairy fouling, electrochemistry, microelectrodes, square wave voltammetry

## Abstract

Fouling in food processing environment can cause the increase of production costs due to additional cleaning steps and risk of contamination of food products. There is a demand to introduce advanced techniques to detect fouling in food processing equipment. Cyclic voltammetry (CV) and square wave voltammetry (SWV) were probed in this work to detect the dairy fouling and the reconstructed dairy emulsion by platinum‐based interdigitated microelectrodes. The results demonstrated that both methods can potentially be used for the fouling detection, since the attachment of fouling to the microelectrode surface leads to lower current responses compared to the clean microelectrodes.

## Graphical abstract

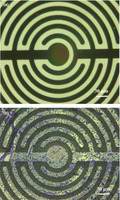

## 1 INTRODUCTION

Industrial food processing equipment is highly challenged by fouling formation on the inner wall of equipment during operation. The presence of fouling leads to the significant decrease of the equipment's performance. In industrial processing equipment, different types of fouling can be found (Groysman, 2017; Kazi, 2012), which can be classified according to the type of equipment undergoing fouling; to the type of fluid causing the fouling; or to the key chemical/physical mechanism giving rise to the fouling. According to Groysman, 2017, fouling types can be divided into the following categories according to the mechanisms: crystallization fouling, particulate fouling, chemical reaction fouling, corrosion fouling, and biofouling. In these categories (except for biofouling), the fouling layer is formed from deposited accumulations of nonmicrobiological origin. In biofouling, the accumulations refer to the attachment of microorganisms to the surfaces, which can grow when nutrients are available (Bhushan, 2016). In the literature, such attachments of microorganisms are also called a biofilm.

The mechanisms of fouling and biofouling attachment are different. In fact, fouling is often a precursor for biofouling due to the reduced heat transfer rate (Ibrahim, 2012). Prior to bacterial adhesion, organic and inorganic molecules from milk can adsorb to surfaces and form a first fouling layer. Thus, the concentration of nutrients closer to the surface is higher than that of the liquid phase. In addition, the fouling layer can change the physical properties of the food contact surface, leading to the increase of the bacterial attachment and subsequently to the formation of biofouling (Fratamico, Annous, & Gunther, 2009). Biofouling increases the risk for microbial contamination and can be a reason for limiting the run length in manufacturing plants (Bhushan, 2016; Brooks & Flint, 2008; Chmielewski & Frank, 2003). Therefore, it is important to detect fouling on different surfaces in order to decrease the risk of microbiological contamination of processed products due to possible biofouling formation.

Fouling formation in the food processing equipment involves the deposition from liquids and suspension of liquid in liquids or solids in liquids. In the dairy processing equipment, particularly in manufacturing processes at lower temperatures, the fouling chemical reaction can be found most often. Conversely, at higher temperatures, crystallization fouling is most prevalent. Components that are mainly responsible for fouling in dairy processing equipment are calcium phosphate and whey proteins, particularly β‐lactoglobulin (β‐LG; Visser & Jeurnink, 1997). According to the literature (Kessler, 2006), there are two types of dairy fouling: Type A and Type B. Fouling formation caused by thermally unstable proteins is dominant at lower temperatures (70ºC–90ºC) and consists of about 60% protein and 40% minerals (Type A). Fouling due to the precipitation of calcium phosphate is dominant at high temperatures (>110ºC) and consists of less than 20% protein and up to 80% minerals, of which more than 80% is calcium phosphate (Type B).

It was described that the first step in dairy fouling formation is the adsorption of a monolayer of proteins onto the surface that occurs already at room temperature. The formation of macroscopic layers of fouling follows in a next step by particle formation (whey protein aggregates and calcium phosphate) in the bulk of the liquid being processed at higher temperatures. Many studies to explore the process of fouling have been reported by many researchers. However, a complete understanding of fouling mechanisms remains elusive due to the complexity of the dairy systems (Visser & Jeurnink 1997).

Monitoring devices for the detection of fouling‐relevant parameters are classified into three levels of information provided by these devices (Flemming, 2011). Level 1 monitoring methods, for example, friction resistance measurement, heat transfer resistance measurement, quartz crystal microbalance, surface acoustic waves, and differential turbidity measurement, provide information about the kinetics of deposition formation and changes of thickness, but cannot differentiate between microorganisms and abiotic deposit components. Level 2 monitoring methods, for example, FTIR‐ATR spectroscopy and microscopical observation, can distinguish between biotic and abiotic components of a deposit. Level 3 monitoring methods, for example, FTIR‐ATR spectroscopy in a flow‐through cell and NMR imaging of deposits in pipes or porous media, provide detailed information about the chemical composition of the deposit (Flemming, 2011). It is important to note that in real industrial processes, not all monitoring techniques are implemented in daily operations. On the one hand, there are a few commercial methods to detect fouling formation in industrial equipment. For example, heat transfer changes or increase of the frictional pressure drop can be measured (Characklis et al., 1990; Lee et al., 1998). On the other hand, a major drawback of both methods is a lack of information on the extent and location of fouling. Although these techniques provide fast information on fouling formation without sampling, these methods are not suitable for the detection of fouling at the early stages of formation. According to Characklis et al., 1990, there are only significant changes in the friction factor when the biofouling reaches a critical thickness of about 35 µm. There are also other methods that provide a higher sensitivity of fouling detection, but they are less common in industrial equipment. For example, optical sensors analyze the relation between emission, transmission, and absorption of different light sources. Fouling can be detected with optical sensors, since it can act as an additional refractor (Fischer, Triggs, & Krauss, 2015). Also, mass‐sensitive sensors such as quartz crystal microbalance (QCM) devices were used in real‐time biofilm monitoring and showed promising results (Olsson, Mitzel, & Tufenkji, 2015; Tam et al., 2007). The resonance frequency of the quartz depends on the adsorbed mass upon it, and thus, the mass variation per unit can be measured. However, QCM devices cannot differentiate between different fouling types (mineral fouling, particle fouling, organic fouling, and biofouling (Epstein, 1981)).

Lastly, some studies about electrochemical devices have been published to explore the fouling detection in industrial equipment (Kang, Kim, Tak, Lee, & Yoon, 2012; Muthukumaran, Jagadeesh, & Srividhya, 2016; Pavanello et al., 2011). According to the IUPAC definition, electrochemical devices transform the effect of an electrochemical interaction between analyte and electrode into a useful signal, which can be subdivided into voltammetric sensors, potentiometric sensors, chemically sensitized field‐effect transistor (CHEMFET), potentiometric solid electrolyte gas sensors, and impedance sensors as well (Hulanicki et al., 1991). For example, voltammetric sensors are the most prevalent in biofilm detection (Ahmed, Rushworth, Hirst, & Millner, 2014). In voltammetry, information about an analyte is obtained by measuring the current as a function of the varied potential. This technique is based on the measurement of the current resulting from the electrochemical oxidation or reduction of an electroactive species. The resulting current is directly correlated with the bulk concentration of the electroactive species or its production or consumption rate within the adjacent biocatalytic layer (Thévenot, Toth, Durst, & Wilson, 2001). Cyclic voltammetry (CV) is a quantitative electrochemical method used to study oxidation or reduction peaks, which are proportional to the concentration of a chemical species that oxidizes or reduces the electrode. In CV, the potential sweep is cycled over time. The potential cycling enables the visualization of the forward and backward reactions (Bard & Faulkner, 2001). CV can be used to identify the potentials at which active redox couples are oxidized and reduced and are an electrochemical technique capable of monitoring redox reactions (Lewandowski & Beyenal, 2013). Square wave voltammetry (SWV) is a pulse technique in which symmetrical square pulses at progressively increasing potentials are applied to an electrochemical system. A square wave is superimposed on a staircase ramp (Bard & Faulkner 2001; Ozkan, Kauffmann, & Zuman, 2015).

In order to conduct electrochemical measurements, the electrochemical sensor is placed in an electrochemical cell, which involves the presence of a faradaic current. The electrode where the oxidation takes place is named anode, and the cathode is the electrode where the reduction takes place, respectively. Anodic reactions generate electrons, whereas electrons are accepted in cathodic reactions (Bagotsky, 2005; Bard & Faulkner 2001; Bard, Inzelt, & Scholz, 2012).

A clear trend in the decrease of the size of electrochemical electrodes can be observed in recent decades. An example is the reduction of the thickness of electrochemical electrodes to the nanosize range. The response time of such electrodes is shorter compared to classical electrodes (Gründler, 2007). Electrochemical interdigitated microelectrode have been used for glucose (Huang et al., 2014) and biofilm detection (Becerro et al., 2015; Strycharz et al., 2011; Tubia, Paredes, Pérez‐Lorenzo, & Arana, 2018). Interdigitated microelectrode arrays consist of a series of parallel microband electrodes in which alternating microbands are connected together (Varshney & Li, 2009). Also, interdigitated microelectrodes have an improved and increased signal‐to‐noise ratio (Min & Baeumner, 2004). According to IUPAC, microelectrode arrays can be prepared by dispersing sufficiently small particles of a conductive material in an insulator or by photolithography (Stulik, Amatore, Holub, Marecek, & Kutner, 2000).

Many studies describe the electrochemical detection of a microbiologically cased deposit, called biofouling or biofilm, particularly due to the extracellular electron transfer of microorganisms. In the dairy industry, however, fouling of nonmicrobiological origin can be a precursor of microbiological contamination of the processing equipment. Thus, dairy fouling should be detected at an early stage of adhesion. However, very few publication can be found in literature that discusses the detection of dairy fouling using electrochemical techniques by interdigitated microelectrodes. Thus, this study aimed to prove the usability of electrochemical techniques, namely cyclic voltammetry (CV) and square wave voltammetry (SWV), to detect dairy fouling. The changes in current between the clean microelectrodes and the microelectrodes with attachment dairy fouling were determined. Surface of the microelectrodes after the fouling attachment was shown using microscopy.

## 2 MATERIALS AND METHODS

### 2.1 Chemicals

Potassium ferrocyanide 1% was obtained from Clin‐Tech (Guildford, United Kingdom*).* Potassium chloride 3 mol/L and crystal violet solution 1% were supplied by Merck (Darmstadt, Germany). Ethanol 70% and meet extract were obtained from VWR (Darmstadt, Germany). Powdered skimmed milk was purchased from Nestlé Deutschland AG (Frankfurt, Germany).

### 2.2 Sample preparation

A reconstituted dairy emulsion was used for the formation of dairy fouling. The dairy emulsion was reconstituted by diluting 2.5 g powdered skimmed milk in 100 ml demineralized water at 22.5ºC ± 0.5ºC. Water activity (a_w_) of powdered skimmed milk was 0.243 ± 0.032. The dairy emulsion was stirred for 5 min at approximately 120 rpm to achieve homogeneous dilution. The prepared dairy emulsion contained 0.023 g 100 ml**^−1^** of fat, 1.3 g 100 ml**^−1^** of carbohydrates, 0.9 g 100 ml**^−1^** of proteins, and 0.028 g 100 ml**^−1^** of salt. For incubation, the 6‐well plates were used (Greiner Bio‐One GmbH, Frickenhausen, Germany). After cleaning and activation, the microelectrodes were immersed into wells. Then, 5 ml of the prepared dairy emulsion was added to each well. The 6‐well plates with microelectrodes and dairy emulsion were incubated for 18 hr at 30°C in the climate chamber. After 18‐hr incubation, the microelectrodes were removed from wells. Then, the microelectrode was used for electrochemical measurements. Both the dairy fouling that was formed of the microelectrode surface and dairy emulsions were measured and compared with the reference, the nonincubated dairy emulsion. Three types of samples were measured: (1) the reconstituted dairy emulsion after 18 hr of incubation, (2) the dairy fouling covering the microelectrode immersed in the reconstituted dairy emulsion after 18 hr of incubation, and (3) the nonincubated reconstituted dairy emulsion as the reference. Before each measurement, 5 µl of either the nonincubated reconstituted dairy emulsion or the reconstituted dairy emulsion after 18 hr of incubation was pipetted onto the surface of the microelectrodes.

### 2.3 Electrochemical setup

For the experiments, a drop cell and interdigitated microelectrodes (MicruX Fluidic, Oviedo, Spain) with four electrodes (one auxiliary, one reference electrode, and two working electrodes) were used. The working electrode has 120 feet pairs, which are 10 µm wide, with a 10 µm gap between each foot. The diameter of the electrochemical cell is 2.0 mm. A PalmSens BV bipotentiostat was used to run measurements. The measurements were performed at temperature 22.5ºC ± 0.5ºC.

Before each use, the microelectrodes were cleaned using demineralized water and ethanol 70%. Then, the microelectrodes were activated electrochemically with 10 µl of 0.1 M potassium chloride by cyclic voltammetry (12 cycles between −1.5 and 1.5 V, scan rate 100 mV/s). After activation, the microelectrodes were cleaned using sterile demineralized water.

### 2.4 Voltammetric methods

In this work, cyclic voltammetry and square wave voltammetry were probed. In cyclic voltammetry, three cycles were applied to each microelectrode with the following parameters: E_begin_ = −0.5 V, E_vtx1_ = −0.5 V, E_vtx2_ = 1.0 V, and scan rate = 0.050, 0.100, and 0.250 V/s with overall duration of 255, 135, and 63 s, respectively. In the square wave voltammetry, the following settings were selected: E_begin_ _=_ −0.5 V, E_end_ = 0.05 V, E_amplitude_ = 0.024 V, and frequency = 25, 50, and 100 Hz with overall duration of 54, 35, and 25 s, respectively. In SWV measurements, anodic current was measured. The measurements were done in triplicate. Three scans were conducted during one measurement. A detailed description of the measurement methodology is shown in Figure Sa and Sb.

### 2.5 ATP bioluminescence

The quantitative detection of the intra‐ and extracellular adenosine triphosphates (ATP) as well as adenosine monophosphate (AMP) was performed using an ATP bioluminescence method. The ATP bioluminescence method is a widespread biochemical technique to detect organic residues, like bacteria or food residues on surfaces and in liquids (Bottari, Santarelli, & Neviani, 2015). The method can be used to rapidly evaluate food and microbial residues on product contact surfaces. The energy stored within the ATP molecule is released as light by splitting of ATP to AMP (adenosine monophosphate). The light can be measured quantitatively and corresponds with the ATP level. The reaction uses the enzyme luciferase. The ATP bioluminescence test was carried out using the ATP + AMP Hygiene Monitoring test kit. The test kit was obtained from Kikkoman Corporation and included the Lumitester^TM^ PD‐30 & LuciPac^TM^ Pen. For liquid samples, the swab stick was removed from its casing and soaked in reconstructed dairy emulsion. For microelectrode surfaces, the wetted swab stick of the LuciPac^TM^ pen was wiped over the surface. Then, the stick was returned to the casing. The test tubing was shaken until the powdery luminescent reagent was dissolved completely. After this step, the swab stick was inserted in the device and the measurement was carried out within 10 s. The results are expressed in relative light units (RLU).

### 2.6 Microscopy

A digital microscope (VHX 100, Keyence, Japan) with bright‐field illumination was used to obtain microscopy images. Before taken images, the microelectrodes were dried for 2 − 3 min at room temperature. To improve the visibility of dairy fouling, 5 µl of crystal violet solution (0.01 g 100 ml^−1^) was used for staining.

## 3 RESULTS

### 3.1 Cyclic voltammetry

**Figure 1:**
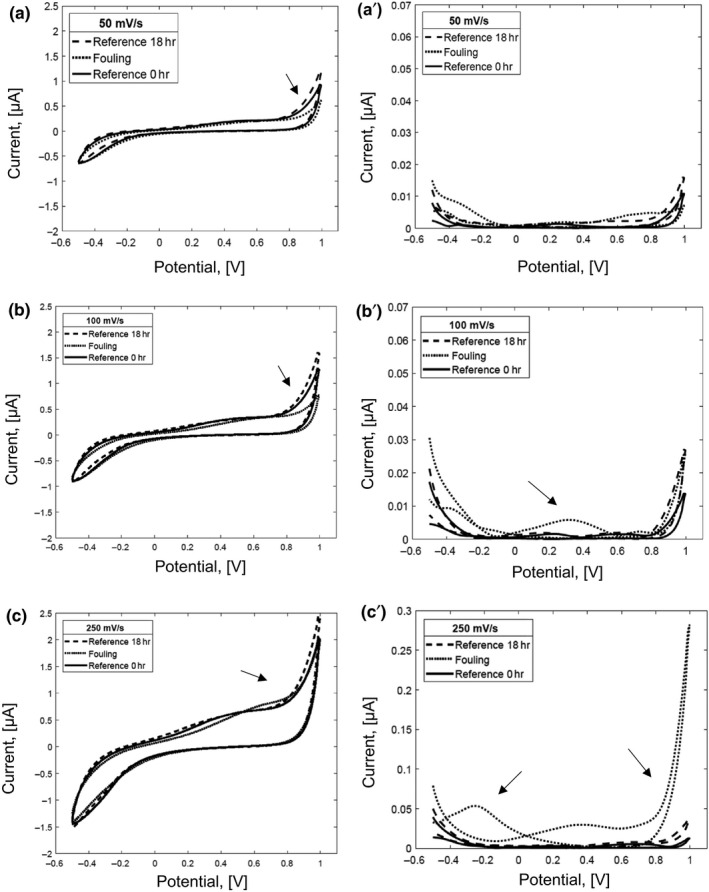
The changes in cyclic voltammograms obtained for (1) reconstituted dairy emulsion after incubation for 18 hr (Reference 18 hr), (2) dairy fouling after incubation for 18 hr (fouling), and (3) nonincubated reconstituted dairy emulsion (Reference 0 hr) at scan rates of (a) 50 mV/s, (b) 100 mV/s, and (c) 250 mV/s with the standard errors of the mean (SE; a´, b´, c´; *i* = 3, *n* = 9)

The cyclic voltammograms at scan rates of 50 mV/s, 100 mV/s, and 250 mV/s of (a) the reconstituted dairy emulsion after 18 hr of incubation, (b) the dairy fouling covering the microelectrode with the reconstituted dairy emulsion after 18 hr of incubation, and (c) the nonincubated reconstituted dairy emulsion as the reference are depicted in Figure 1a‐c, respectively.

The cyclic voltammograms shown in the curves of Figure 1 illustrate that the current signals of anodic (oxidation) and cathodic (reduction) curves increase with an increasing scan rate for all samples. The reconstituted dairy emulsion after 18 hr of incubation illustrates the highest current response in the anodic (oxidation) curve, followed by a nonincubated reconstituted dairy emulsion and finally by dairy fouling. This succession remains at all three scan rates. The differences between the samples are less pronounced for the cathodic curves that for the anodic curves.

It can be seen that the errors from the dairy fouling and the incubated reconstituted dairy emulsion are higher than the errors from the reference at scan rates of 100 mV/s and 250 mV/s (Figure 1b´, c´). This behavior can be explained by the nonhomogeneous surface coverage of fouling of the working electrode (Figure 4b) and to the structural changes of the dairy emulsion during the incubation time. The highest standard error of the mean is found at the potentials where the anodic and the cathodic curves have a peak (Figure 1a‐c). The highest standard error of the mean is obtained for the dairy fouling measured at the scan rate of 250 mV/s.

Table 1 summarizes the anodic and cathodic peak currents as well as the peak potentials for the investigated samples. From the dairy fouling voltammograms, an anodic peak at potentials of 0.38 V and 0.88 V is recorded at scan rates of 100 mV/s and 250 mV/s, respectively. A cathodic peak is found for the dairy fouling at –0.38 V and –0.36 V at a scan rates of 100 mV/s and 250 mV/s, respectively (Figure 1 and Table 1). The vertical distance between forward and reverse scans increases at higher scan rates. With increasing scan rate, the curve shapes appear wider and more distinctive. Notable differences between the reconstituted dairy emulsion 18 hr and dairy fouling are found in the anodic curve in the potential window 0.7–1.0 V while in the cathodic curve from –0.2 to –0.5 V (Figure 1).

**Table 1:** Anodic (oxidation) and cathodic (reduction) peak currents (μA) and peak potentials (V) of the (1) reconstituted dairy emulsion after incubation for 18 hr, (2) dairy fouling, and (3) nonincubated reconstituted dairy emulsion obtained from the cyclic voltammetry results (*i* = 3, *n* = 9)

Scan rate (mV/s)	I_pA_, I_pC_ (μA) E_pA_, E_pC_ (V)	Nonincubated reconstituted dairy emulsion	Dairy fouling	Reconstituted dairy emulsion 18 hr
50	I_pA_	0.10 ± 0.07	0.03 ± 0.00	None
	E_pA_	0.45 ± 0.01	0.54 ± 0.01	None
	I_pC_	−0.04^a^	None	None
	E_pC_	−0.43^a^	None	None
100	I_pA_	0.06 ± 0.00	0.06 ± 0.02	0.03 ± 0.00
	E_pA_	0.42 ± 0.01	0.58 ± 0.04	0.38 ± 0.05
	I_pC_	−0.18^a^	−0.68^a^	−0.21 ± 0.14
	E_pC_	−0.43	−0.49^a^	−0.38 ± 0.08
250	I_pA_	0.13 ± 0.01	0.07 ± 0.04	0.07 ± 0.01
	E_pA_	0.44 ± 0.00	0.56 ± 0.05	0.88 ± 0.02
	I_pC_	None	−0.13 ± 0.08	−0.07 ± 0.01
	E_pC_	None	−0.47 ± 0.01	−0.36 ± 0.02

a Only an average of one sample could be evaluated (1 sample—3 scans).

Table 1 illustrates that the current at anodic peaks increases for the dairy fouling with enhanced scan rate. The potential of the anodic and cathodic peaks for the dairy fouling moves toward positive potentials with enhanced scan rate. The result assumes that an irreversible process takes place in the electrochemical cell. For both reconstituted dairy emulsions, no considerable differences in peak potential are observed with increasing scan rate.

**Figure 2:**
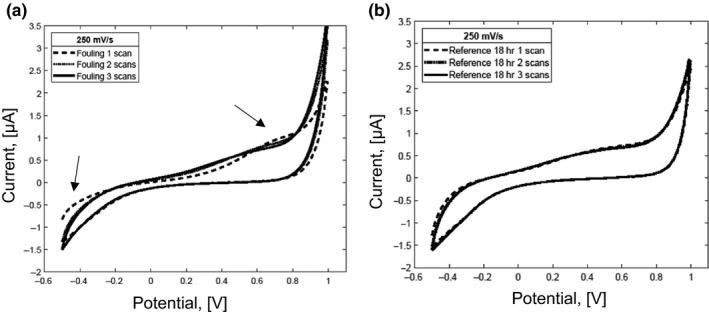
Cyclic voltammograms (3 scans) for dairy fouling 18 hr (a) and reconstituted dairy emulsion 18 hr (b) at scan rates of 250 mV/s

Figure 1c´ shows the highest values of the standard error of the mean for the dairy fouling at scan rate of 250 mV/s. It can be explained that the fouling could be detached from the surface of the microelectrode after each measurement, which could have led to the increase in fouling on the surface of the working electrode. As an example, the cyclic voltammograms with three consecutive scans, of the dairy fouling and the reconstituted dairy emulsion after 18 hr of incubation at scan rate of 250 mV/s, are shown in Figure 2a,b, respectively. The results of the dairy fouling measurements illustrate the differences between scans (Figure 2a). On the contrary, the measurements of the reconstituted dairy emulsion after 18 hr do not show differences between three consecutive scans (Figure 2b).

Figure 2b shows that the current of both the anodic (oxidation) and cathodic (reduction) peaks for the incubated dairy emulsion does not change after a few consecutive scans. On the contrary, in Figure 2a the changes in the cathodic (reduction) and anodic (oxidation) curves for the dairy fouling are evident. It was published that for a *P. fluorescens* aggregation on electrodes, the pattern of the voltammograms obtained after 1, 2, 5, 10, and 100 scans approximated the voltammogram obtained for a surface without biofilm (Vieira, Pinho, Gião, & Montenegro, 2003). The reported finding was similar to results observed in the study of Vieira et al., 2003, as shown in Figure 2b. According to Vieira et al., 2003, the distance between the deposited bacteria and the surface is very short that, according to DLVO theory, is caused due to an interaction on the primary minimum of energy (Hori & Matsumoto, 2010)). It means that the redox reactions cannot take place on specific places of the electrode. Once the potential is applied, the distance between the bacterial aggregations and the surface may enlarge. Then, redox reactions may occur on the electrode surface that was covered by deposited bacteria. In this case, a voltammogram similar to the voltammogram of a surface without deposit is obtained. It suggests that similar behavior may be also observed for the dairy fouling.

Table 2 shows the total sum of the standard error of the mean (*SSE*) and the sum of the variances (*SV*) for (1) the reconstituted dairy emulsion before incubation, (2) the dairy fouling, and (3) the reconstituted dairy emulsion after 18 hr of incubation at scan rates of 50 mV/s, 100 mV/s and 250 mV/s.

**Table 2:** The sum of the standard error of the mean (*SSE*) and the sum of the variances (*SV*) of cyclic voltammetry measurements at scan rates of 50 mV/s, 100 mV/s, and of 250 mV/s (*p* < .05)

Scan rate (mV/s)	Nonincubated reconstituted dairy emulsion	Dairy fouling	Reconstituted dairy emulsion 18 hr
50			
*SSE*	0.315	0.754	0.646
SV	0.011	0.036	0.032
100			
*SSE*	0.551	1.375	0.799
SV	0.031	0.136	0.077
250			
*SSE*	1.165	10.132	2.162
SV	0.127	8.231	0.313

As expected, the reconstituted dairy emulsion before and after incubation shows the lowest sum of the standard error of the mean and the sum of the variances. On contrary, the dairy fouling has the highest sum of the standard error of the mean and the sum of the variances. It can be attributed to the nonhomogeneous biofouling attachment to the surface of the microelectrodes (Figure 4b). For all the samples, a clear relation between the scan rates and the sum of the standard error of the mean or the sum of the variances is found. The lowest errors are obtained at a scan rate of 50 mV/s, while the highest are obtained at a scan rate of 250 mV/s.

### 3.2 Square wave voltammetry

**Figure 3:**
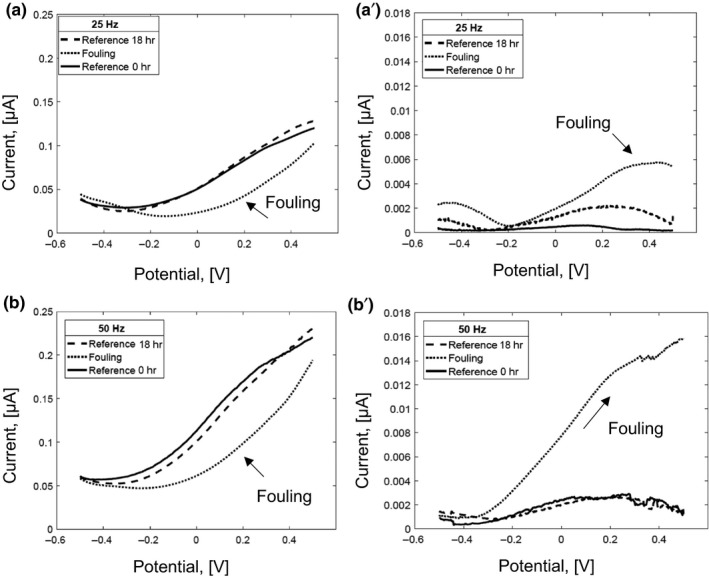
Square wave voltammograms (forward scan) for (1) reconstituted dairy emulsion after incubation for 18 hr (Reference 18 hr), (2) dairy fouling 18 hr (fouling), and (3) nonincubated reconstituted dairy emulsion (Reference 0 hr) at frequencies of 25 Hz (a) and 50 Hz (b) with the respective standard errors of the mean (SE; a´, b´; *i* = 3, *n* = 9)

The voltammograms of SWV measurements obtained at frequencies of 25 Hz and 50 Hz for (a) the reconstituted dairy emulsion after 18‐hr incubation, (b) the dairy fouling covering the microelectrode with the reconstituted dairy emulsion after 18‐hr incubation, and (c) the nonincubated reconstituted dairy emulsion as the reference are plotted in Figure 3.

The curves in Figure 3 show that, similarly to the results of cyclic voltammetry, the current increases with increasing frequency. The highest currents are found for the reconstituted dairy emulsions before and after incubation, followed by the dairy fouling. It can be assumed that an additional fouling layer on the working electrode may cause a resistance that lowers the current response. The standard error of the mean for the dairy fouling is much higher compared to the errors obtained for the incubated and nonincubated dairy emulsions. Moreover, the standard errors of the dairy fouling mean increased with the increasing scan rate. The measurements were also conducted at frequency of 100 Hz. However, it was not possible to conduct the evaluation of the obtained data due to the high noise level.

Table 3 shows the total sum of the standard error for (a) the reconstituted dairy emulsion before incubation, (b) the dairy fouling covering the microelectrode with the reconstituted dairy emulsion after 18‐hr incubation, and (c) the reconstituted dairy emulsion after 0 hr incubation at frequencies of 25 and 50 Hz.

**Table 3:** The sum of the standard error (*SSE*) of the mean and the sum of variances (*SV*) of square wave voltammetry measurements at frequencies of 25 Hz and 50 Hz

Frequency (Hz)	Nonincubated reconstituted dairy emulsion	Dairy fouling	Reconstituted dairy emulsion 18 hr
25			
*SSE*	0.310	2.742	1.131
SV	0.001	0.100	0.016
50			
*SSE*	1.621	7.330	1.596
SV	0.031	0.759	0.028

Table 3 shows that the sum of the standard error of the mean and the sum of the variances increased for all samples with increasing frequency. The highest sum of the standard error of the mean and the sum of the variances among were found for the dairy fouling. Analogical results were also found for the results of cyclic voltammetry measurements (Table 2).

### 3.3 ATP bioluminescence measurement

The mean value of the ATP bioluminescence test for the nonincubated reconstituted dairy emulsion was 6,396 ± 851 RLU (*n* = 9). After 18‐hr incubation, the RLU value slightly increased to 7,386 ± 159 RLU (*n* = 9). The RLU value of clean electrode surface without dairy fouling was 14 ± 6 RLU (*n* = 9). The RLU value of the electrode surface increased to 193 ± 60 RLU after 18 hr of incubation in the reconstituted dairy emulsion. In dairy industry, the method detects food/organic residues and offer proactive cleanliness management. Since the ATP bioluminescence test can detect all organic matter on food contact surfaces (Costa, Andrade, Soares, Passos, & Brandão, 2006), the obtained results confirm that initial whey protein adhesion to microelectrode occurred.

### 3.4 Microscopy

**Figure 4:**
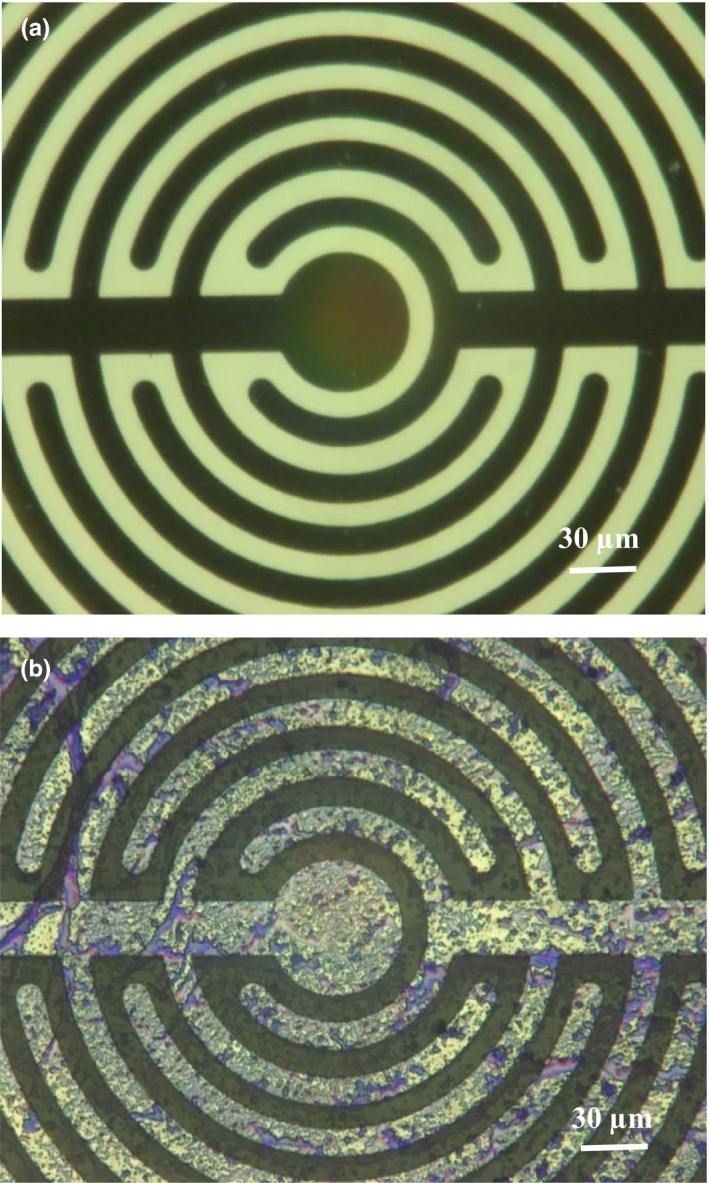
Micrographs of the clean interdigitated microelectrode (a), the interdigitated microelectrode surface with attached dairy fouling (18‐hr incubation at 30°C) (b). All the samples were recorded with a digital microscope (VHX 100 Microscope) at 500 × magnification

Figure 4a,b shows the clean microelectrode and the dairy fouling on the microelectrode surface stained with crystal violet solution (0.01 g/100 ml).

Figure 4b indicates that the fouling attachment on the microelectrode surface was not homogeneous with thicker and thinner clusters. It is assumed that nonhomogeneous distribution of the dairy fouling (Figures 1 and 2) may lead to the large standard deviations.

## 4 DISCUSSION

Cyclic voltammetry and square wave voltammetry were used to determine dairy fouling on interdigitated ring array microelectrodes.

When discussing the attachment of fouling to the contact surfaces, the initial composition of fouling has to be considered. Since there are different components involved in fouling formation, the first question is what will attach first to the contact surface: proteins or minerals. According to literature, whey protein denaturation and aggregation as well as calcium phosphate particle formation are two different processes and both processes follow different kinetics. Belmar‐Beiny & Fryer, 1993 reported that, for a solution of a whey protein concentrate, the first layer of the deposit contains mainly proteins. This indicates that the first layer adsorbed at room temperature will always be proteinaceous. The deposition of aggregates, calcium phosphate and whey protein particles, will occur at higher temperatures after the surface has been covered by a protein monolayer (Visser & Jeurnink 1997).

Indeed, β‐lactoglobulin (β‐LG) and ɑ‐lactalbumin (ɑ‐LA), which are responsible for initial fouling formation, are of a globular nature and both proteins are act on heat. β‐Lactoglobulin is the dominant protein and also more heat sensitive than ɑ‐lactalbumin. In literature, it was shown that the initial binding of β‐LG to a metal surface is due to a chemical linkage (Visser & Jeurnink 1997). The electrochemical study of Roscoe, Fuller, & Robitaille, 1993 showed that at pH = 7.0 and 26°C, β‐LG adsorbs onto a platinum surface as a monolayer by binding its carboxyl groups to a platinum surface OH group under decarboxylation. It was reported that the first layer of fouling can be formed at room temperature. However, the first layer formed at room temperature cannot cause a further growth of protein fouling due to the fact that bulk fouling starts at temperatures above 72°C (Roscoe et al., 1993).

The cyclic voltammetry measurements of each sample showed that the current responses and the peak current magnitude increased with an increasing scan rate. Interdigitated ring array microelectrodes covered by dairy fouling showed the lowest current responses, when compared with the incubated and nonincubated dairy emulsions. This observation indicates that fouling on the working electrode represents an additional resistance, which extenuates the current response (Becerro et al., 2015). It was reported that the adsorption of β‐lactoglobulin (β‐LG) and ɑ‐lactalbumin (ɑ‐LA) to the surface enhanced the decrease of the current (Roscoe et al., 1993). This agrees with literature reporting that the surface coverage of the working electrode affects the current response (Bard & Zoski, 2000). In the study of Rana et al., 2018, it was found that with increasing concentrations of Zn acetate in milk, the current decreased. The authors of the study (Rana et al., 2018) suggested that these metal ions can be attached to the active sites of proteins and reduce the flow of electrons onto the working surface of the electrode, thus decreasing the peak current.

The study of Rouhana, Budge, Macdonald, & Roscoe, 1997 investigated the adsorption of ɑ‐lactalbumin and bovine serum albumin using cyclic voltammetry. The interfacial behavior of proteins was studied on a platinum electrode over a temperature range from 0ºC to 90ºC. It was reported that both proteins adsorbed strongly on the metal surface with increasing temperature. Mixtures of the two proteins showed substantial increase in the surface charge density at the higher temperatures of 80ºC and 90ºC. The authors suggested that this may be caused by intermolecular interaction which, in more concentrated solutions, provides a strong gel network. The interactions between protein and a metal surface, resulting in adsorption, can be affected by the protein properties, including other parameters such as pH, concentration, and temperature (Kiss, 1993).

In square wave voltammetry, the current responses and the peak current magnitude of the voltammograms increased with the increasing frequency. As obtained in CV, microelectrodes covered by the dairy fouling showed the lowest current responses within their substance groups. Similarly to CV results, the adsorption of proteins to the surface can lead to the current's decrease. However, SWV was a less suitable method for the purpose of distinguishing dairy fouling from their corresponding substances with the chosen parametric setup. No oxidation peak currents could be detected. Only the current signal height could be used for the comparison between samples. This can be explained by the fact that SWV is better for quantitative analysis and therefore more sensitive in detecting specific analytes (Mirceski, Guziejewski, & Lisichkov, 2013). SWV usually provides less distortion, which makes fitting of data to theoretical models more accurate. According to the literature, SWV is more suitable for the detection of low concentrations of electrochemically active species, which cannot be detectable by other voltammetric techniques. SWV can be recommended for analytical purposes, however, for which frequencies of 100 Hz might be too high (Mirceski, Komorsky‐Lovric, & Lovric, 2007). Similar result was also obtained in this work. At lower frequencies, the duration of the measurement is much longer and this can lead to structural changes of the tested sample. This result agrees with the study (Nguyen, Renslow, Babauta, Ahmed, & Beyenal, 2012), where, as the frequency was increased from 1 to 40 Hz, the flavin redox peak centered around −425 mV Ag/AgCl increases. Moreover, when the frequency was greater than 20 Hz, the noise level became significant, a reproducible effect across multiple flavin microelectrodes; thus, 20 Hz was chosen as the optimum frequency. Therefore, it is doubtful whether SWV is suitable for industrial implementations.

Also, emulsion, for example, dairy emulsions as measured in this work, is of importance and cyclic voltammetry allows them to be investigated. Through electrochemical investigations, the complex structure of dairy emulsions as mixture of immiscible liquids should be considered. According to Bagotsky, 2005, processes that may need to be considered in these multiphase liquids are (a) partitioning of solutes, (b) electro‐dissolution of one phase into the other, (c) the electrochemical conversion of a solute in phase to another redox level accompanied by ion exchange, and (d processes involving material adsorbed at the interface between the two phases. In voltammetrical studies of stabilized microemulsions, processes monitored were shown to be consistent with reactions in which the reactant diffused from the emulsion droplet toward the electrode surface (Bagotsky, 2005). Also, the adsorption of milk proteins at interfaces provides information on the properties of dairy emulsions (Dickinson, 1989).

The presence of fouling is often assumed when poor process performance and decreased product quality are observed after production. Moreover, according to literature there is a lack of a reliable monitoring and detecting sensor system that allows to monitor both the fouling development and the cleaning processes in food processing equipment (Marchand et al., 2012). Real‐time detection and monitoring of fouling would ensure better quality control of dairy products during processing in dairy industry. In addition, the monitoring of the cleaning processes would be advantageous for the development of intelligent cleaning processes. The use of electrochemical interdigitated microelectrodes for the detection of biofilms and CIP cleaning solutions has been already reported in the previous study (Fysun, Khorshid, Rauschnabel, & Langowski, 2019). However, when working with electrochemical microelectrodes, it also should be taken into account that electrochemical microelectrodes require a direct contact with a target analyte, for example, dairy products being manufactured. Therefore, an implementation of electrochemical microelectrodes into food processing equipment must be retained regarding hygienic design standards of food processing equipment.

## 5 CONCLUSION

The results showed that cyclic voltammetry and square wave voltammetry measurements combined with interdigitated microelectrodes can potentially be used for the detection of dairy fouling. Cyclic voltammetry demonstrated good results for the fouling detection and for the differentiation between the dairy emulsion and the dairy fouling at different scan rates. The attachment of dairy proteins leads to the decrease in the current in cyclic voltammograms, which can be explained by the additional insulated layer on the microelectrodes that extenuates the current response. Square wave voltammetry can be recommended for the fouling detection at low frequencies. In future, electrochemical investigations under flow conditions should be performed. Moreover, the microelectrode integration into specific high‐risk parts of industrial dairy equipment has to be studied.

## CONFLICT OF INTEREST

The authors declare that they have no conflict of interest.
