# Supplementary material for: Detection of dairy fouling by cyclic voltammetry and square wave voltammetry
Source: Food Sci Nutr. 2020 Mar 16;8(7):3070–80. doi: 10.1002/fsn3.1463 (PMC7382167; doi:10.1002/fsn3.1463)
Supplement: Supplementary file 1 [file FSN3-8-3070-s001.docx]

Supplementary material

Detection of dairy fouling by cyclic voltammetry and square wave voltammetry

**Olga Fysun^1,2,†^, Sara Khorshid^2,3, ††^, Johannes Rauschnabel^2^, Horst-Christian Langowski^1,4^**

^1^TUM School of Life Sciences Weihenstephan, Technical University of Munich, Freising, Germany

^2^Robert Bosch Packaging Technology GmbH, Waiblingen, Germany

^3^ Department of Mechanical and Process Engineering, University of Kaiserslautern, Kaiserslautern, Germany

^4^Fraunhofer Institute for Process Engineering and Packaging IVV, Freising, Germany

^†^ Present address: Robert Bosch GmbH, Reutlingen, Germany

^††^Present address: Sanofi-Aventis Deutschland GmbH, Frankfurt, Germany

Applied potential as a function of time for a CV and SVW measurement is shown in Fig. A and B, respectively**.**

**Figure A**

Applied potential as a function of time for a CV measurement, with the initial, switching, and end potentials represented (1, 2, and 3, respectively) the following parameters: E_begin_ = −0.5 V, E_vtx1_ = −0.5 V, E_vtx2_ = 1.0 V, scan rate = 0.050, 0.100, and 0.250 V s^−1^.

**2**

**1**

**Figure B**

Applied potential as a function of time for a SWV measurement, with the initial, and end potentials represented (1, and 2, respectively) the following parameters: E_begin_ = −0.5 V, E_end_ = 0.05 V, E_amplitude_ = 0.024 V, frequency = 25, 50, and 100 Hz.
